# Metagenome-assembled-genomes recovered from the Arctic drift expedition MOSAiC

**DOI:** 10.1038/s41597-025-04525-8

**Published:** 2025-02-04

**Authors:** William Boulton, Asaf Salamov, Igor V. Grigoriev, Sara Calhoun, Kurt LaButti, Robert Riley, Kerrie Barry, Allison A. Fong, Clara J. M. Hoppe, Katja Metfies, Kersten Oetjen, Sarah Lena Eggers, Oliver Müller, Jessie Gardner, Mats A. Granskog, Anders Torstensson, Marc Oggier, Aud Larsen, Gunnar Bratbak, Andrew Toseland, Richard M. Leggett, Vincent Moulton, Thomas Mock

**Affiliations:** 1 School of Computing Sciences, University of East Anglia, Norwich Research Park, Norwich, NR4 7TJ UK; 2 U.S. Department of Energy Joint Genome Institute, Lawrence Berkeley National Laboratory, Berkeley, CA 94720 USA; 3 Department of Plant and Microbial Biology, University of California Berkeley, Berkeley, CA 94720 USA; 4 Alfred Wegener Institute, Am Handelshafen 12, 27570 Bremerhaven, Germany; 5 University of Bergen, Thormøhlens gate 53 A/B, 5006 Bergen, Norway; 6 UiT the Arctic University of Norway, Hansine Hansens veg 18, 9019 Tromsø, Norway; 7 Norwegian Polar Institute, Fram Centre, Hjalmar Johansens gate 14, 9296 Tromsø, Norway; 8 Department of Aquatic Sciences and Assessment, Section for Ecology and Biodiversity, Swedish University of Agricultural Sciences, Uppsala, Sweden; 9 University of Alaska Fairbanks, 1731 South Chandalar Drive, AK 99775 Fairbanks, USA; 10 NORCE Norwegian Research Centre, Nygårdsgaten 112, NO-5008 Bergen, Norway; 11 School of Environmental Sciences, University of East Anglia, Norwich Research Park, Norwich, NR4 7TJ UK; 12 Earlham Institute, Norwich Research Park, Colney Lane, Norwich, NR4 7UZ UK

**Keywords:** Molecular biology, Microbial ecology, Water microbiology, Metagenomics, Microbial ecology

## Abstract

The Multidisciplinary Observatory for Study of the Arctic Climate (MOSAiC) expedition consisted of a year-long drifting survey of the Central Arctic Ocean. The ecosystems component of MOSAiC included the sampling of molecular data, with metagenomes collected from a diverse range of environments. The generation of metagenome-assembled-genomes (MAGs) from metagenomes are a starting point for genome-resolved analyses. This dataset presents a catalogue of MAGs recovered from a set of 73 samples from MOSAiC, including 2407 prokaryotic and 56 eukaryotic MAGs, as well as annotations of a near complete eukaryotic MAG using the Joint Genome Institute (JGI) annotation pipeline. The metagenomic samples are from the surface ocean, chlorophyll maximum, mesopelagic and bathypelagic, within leads and under-ice ocean, as well as melt ponds, ice ridges, and first- and second-year sea ice. This set of MAGs can be used to benchmark microbial biodiversity in the Central Arctic Ocean, compare individual strains across space and time, and to study changes in Arctic microbial communities from the winter to summer, at a genomic level.

## Background & Summary

The Central Arctic Ocean is one of the most understudied biomes on Earth and it is home to a significant amount of microbial diversity relative to its area. Furthermore, the Arctic Ocean is warming at a higher rate than lower latitudes, which has detrimental effects on the diversity of Arctic ocean biomes^1^. Microbes that underpin these biomes play an important role as primary producers and therefore as the base of the marine food web and central for biogeochemical cycles. They are also a source of novel genes, many of which have been of interest to the biotechnology industry^2^. It is therefore important to understand the effect of changing environmental conditions on these organisms including the evolution of their genes and genomes. However, due to the inaccessibility of the Central Arctic Ocean, there is a large gap in our understanding of microbial communities which thrive in this habitat. The MOSAiC expedition provided invaluable opportunities to address this knowledge gap and is the first year-long survey of the Central Arctic Ocean, facilitating sampling throughout the entire Arctic winter.

The MOSAiC expedition (2019–2020) was a Lagrangian drift survey, based around the icebreaker RV Polarstern^3^, which moved from north of the Laptev Sea northwards during leg 1 (19^th^ September to 15^th^ December 2019), before drifting southwards toward the Fram Strait (legs 2 to 4, 15^th^ December 2019 to 12^th^ August 2020) and finally returning to the Central Arctic Ocean (leg 5, 12^th^ August to 12^th^ October 2020) at the end of the expedition. During each leg, metagenomes were collected from both sea ice and pelagic waters, with a minority of samples collected from sediment traps under sea ice. Samples were collected either as part of a core time series, from intense observation periods of opportunistic sampling, or as part of the HAVOC project (Ridges – safe HAVens for ice-associated flora and fauna in a seasonally ice-covered Arctic Ocean), collecting samples from ice-ridges, under-ice water, and from sediment traps beneath sea-ice ridges and level ice^4^.

Sea-ice ridges are characteristic features covering 25 to 45% of the Arctic sea ice area^5^. Ridges are formed by pressure from drifting ice. When ice floes are forced together, they break up and are pushed up and down to form a sail above and a keel below the surface water level. The keel consists of ice blocks separated by voids, described as macroporosity, making up ~15–30% of the volume^6,7^. The voids may be empty (in the sail) or filled with liquid or frozen seawater or meltwater (in the keel). While the bottom of level sea ice is known as an important habitat for Arctic marine biodiversity and activity, much less is known of the life within ridge keel voids which constitute unique habitats and biological hotspots in the Arctic Ocean^8–10^. As ridges are logistically harder to navigate and take samples from, they are relatively understudied compared to level ice. The aim of the HAVOC project was to better understand how ice ridges act as a refuge for microbial biodiversity and activity, and how food web and biogeochemical processes at the ice-ocean interface and the underlying water column differ between ridges and level sea ice^4^.

This study consists of metagenome-assembled-genomes (MAGs) assembled from a pilot sequencing project of 15 metagenomes collected during Leg 2 (15^th^ December to 3^rd^ March) of the voyage in the Arctic winter as part of the core time series, and 58 metagenomes associated with the HAVOC project. Advances in metagenomic sequencing, assembly and binning, have generated a wealth of MAG datasets, even for Arctic environments such as in^11^. However, challenges associated with the assembly and binning of eukaryotes have meant that these generally focus on prokaryotic MAGs. Two exceptions are Duncan *et al*. and Delmont *et al*.^12,13^, which generated 21 and 25 eukaryotic medium and high quality MAGs from Arctic metagenomes respectively (i.e. above 50% completeness). The data presented here includes both prokaryotic and eukaryotic MAGs, using coassembly to improve the coverage of eukaryotes. These MAGs can be used to gain insights into microbial diversity and the metabolic potential of microbiomes during the Arctic winter, and the role of ice ridges in maintaining the microbial biodiversity of the Arctic Ocean.

## Methods

### Sampling

Of the 73 samples presented here, 15 were collected as part of a year-round time-series, during leg 2 of the expedition (between 13 January 2019 and 7 February 2020) as described in Winder *et al*.^14^, and sequenced as part of a pilot sequencing project (hereon called pilot samples). Of these samples, 8 were from pelagic layers and the remaining 7 from sea ice. The pelagic pilot samples were collected using a CTD rosette, on three different days. Sequenced pilot samples from a sampling event on February 6^th^ 2020 consist of 2 co-located samples (i.e. replicates, from the same CTD cast and sampled at the same depth, but from different Niskin bottles) taken from a depth of 20 m, and one sample from a depth of 202 m. One sample was collected on February 7^th^ 2020, at a depth of 4082 m. Further, 2 replicates from a depth of 50 m sampled on January 16^th^ 2020 were sequenced.

Additionally, for each of the two biological replicates, a third technical replicate was generated by pooling remaining material from the two replicate samples. These data are summarised in Supplementary Table 1, with the two samples generated through pooling identified with a sample identifier suffix ‘pool’ (column E).

The remaining 7 pilot samples from sea ice were taken from the first-year and second-year MOSAiC ice-coring sites on the floe, as described in Nicolaus *et al*.^15^ and Fong *et al*.^16^. First and second-year sea-ice chemical and physical properties are available in Oggier *et al*.^17,18^, Lei *et al*.^19^, and properties for snow in Macfarlane *et al*.^20^. Generally, 2–4 cores were collected on a weekly basis, cut in 10 cm sections, except the bottom where two 5 cm thick section were cut, and pooled per section to allow for enough biomass in the DNA samples. For the pilot study, samples per depth interval always represent pools of three individual cores collected after each other in the same location (adjacent within 40 cm). Of these, five samples were collected from different 5–10 cm thick sections of cores from the same coring site on February 3^rd^ 2020 from first-year ice; three from the upper part (20–50 cm from the top), one from the middle section (70–80 cm from the top), and one from the bottom-most section (122 to 127 cm from the top) of the sea ice, i.e. at the sea-ice interface. The remaining two samples were second-year ice, also from the bottom most 5 cm section, i.e. from the sea–ice interface, 1.23–1.28 m and 1.43–1.48 m from the top, collected on January 13^th^ and 27^th^ 2020.

**Fig. 1:**
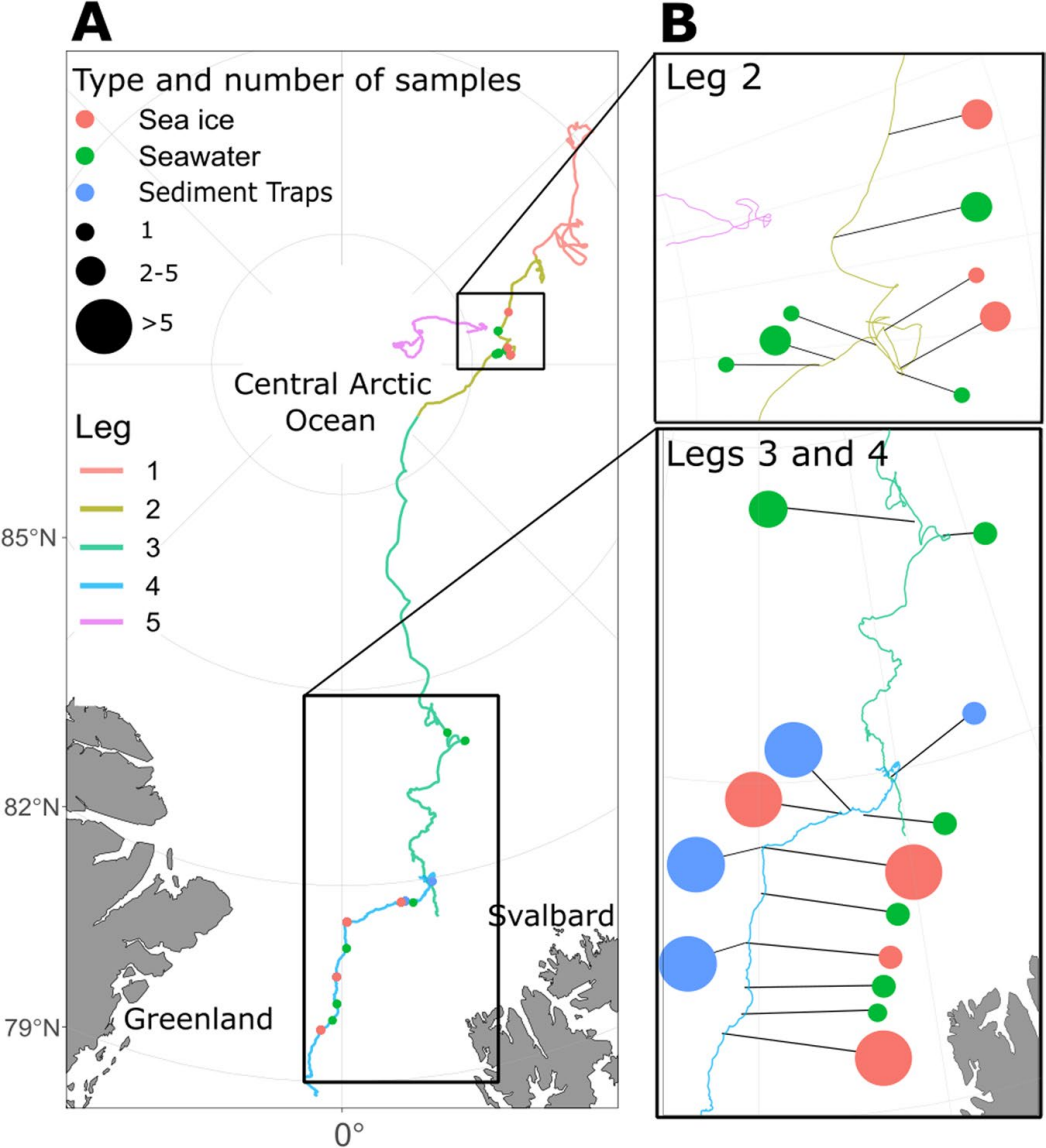
Map showing the locations of the samples. Panel A shows the overall course of the drift, and locations of the samples. Panel B shows more detailed locations, zoomed within the boxes marked in panel A. Samples are from leg 2 (15^th^ Dec. 2019–3^rd^ March 2020), leg 3 (3^rd^ March–6^th^ June 2020), and leg 4 (6^th^ June–12^th^ August 2020), with the drift route generally moving southward from the Central Arctic Ocean. Often, multiple (replicate) samples are co-located, either from the same CTD cast, or as different layers within a single ice core. In panel B the number of co-located samples is represented by the size of the marker.

The 58 metagenomes from the HAVOC project were collected during legs 2, 3 and 4 (collection dates between 22^nd^ January 2020 and 26^th^ July 2020), either: from sediment traps, directly below an ice ridge (7 samples) or level ice (10 samples) at depths of 5, 15 and 50 m, in the water column at 20 m (2 samples), from under ice water below an ice ridge (2 samples) and level ice (3 samples), from seawater taken from voids in the ice ridge (10 samples), or from a 10 cm ice core section at different depths of the ice ridge, as either ridge bottom ice (3 samples), top of void ice (5 samples), bottom of void ice (6 samples), refrozen void ice (4 samples) and ice samples at irregular depths (6 samples). Samples from the same location, depth, and time (Supplementary Table 1) are considered replicates, with samples taken from a total of 24 distinct locations and depths. The number of pooled core sections for each sample, and section thickness, are recorded in Supplementary Table 1. Figure 1 summarises the locations of the samples.

For both the HAVOC and pilot sea ice samples, ice cores were sectioned in the field, transferred to sterile plastic bags, and brought back to the RV Polarstern. On board, 50 ml 0.22 µm filtered sea water was added per cm sea ice, and the sea ice samples melted within 24–36 hours in the dark at around 17–22°C. The use of filtered seawater was made necessary due to logistical constraints during the drift; this is a possible source of contamination for the sea ice samples. For both sea ice and pelagic samples, water was filtered through a Sterivex 0.22 µm filter or when volumes were <500 mL (HAVOC sediment trap samples and three HAVOC ice samples) onto 0.22 µm Durapore filters. The filters were immediately flash frozen in liquid nitrogen and stored at −80 °C on board the RV Polarstern, and subsequently shipped to either the Alfred Wegener Institute (pilot samples), or the University of Bergen (HAVOC samples), at a temperature of −80 °C.

### DNA extraction, purification, and sequencing

Following shipping, DNA from Sterivex filters was extracted using the Qiagen PowerWater DNA kit, following the QIAGEN DNeasy Power Water SOP v1 for the ice and water samples, and the QIAGEN Dneasy Power Soil SOP v1 (QIAGEN N.V., Hilden, Germany) used for the DNA extraction from Durapore filters.

Plates were shipped to the Joint Genome Institute (JGI, CA, USA) under dry ice, and sequenced using 151 base pair paired-end reads with an Illumina NovaSeq S4 device. The library type used was in all cases either the Illumina low or regular concentration protocol, with between 0 and 15 rounds of PCR applied to samples, though in all but 5 cases, this was restricted to only either 0 or 5 rounds.

Supplementary Table 3 outlines the library preparation steps and sequencing protocols used for each of the samples.

### Genome assembly and binning

Samples were first assembled individually using the JGI MAP pipeline^21^, with prokaryotic bins recovered on a per-sample basis. In brief, samples were filtered for quality with BBDuk, error corrected using BBCMS, assembled using SPAdes^22^, and reads mapped back to contigs using BBMap (38.86)^23^. Binning was performed using MetaBAT 2^24^ (v2.1.15) and assessed for quality using CheckM^25^ (v1.1.3). Software and pipeline versions used are listed in Supplementary Table 3.

**Fig. 2:**
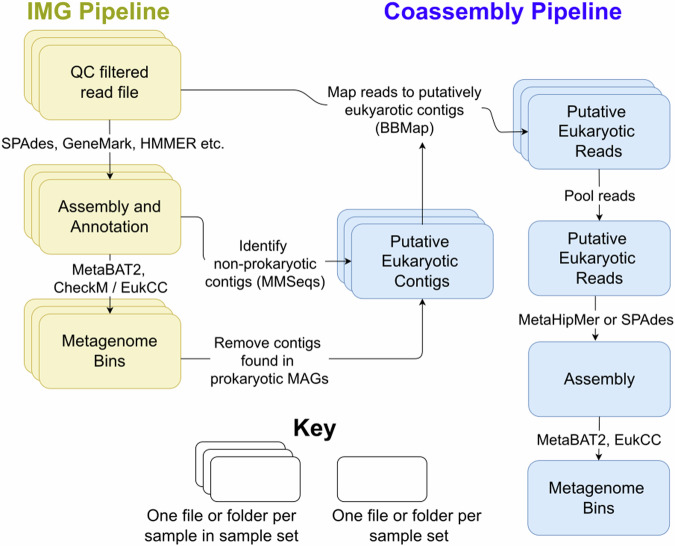
A summary of the IMG metagenome annotation pipeline, and the coassembly pipeline used for the two sample sets; either the pilot samples or the HAVOC samples. Coloured boxes indicate intermediate folders or files, either one per sample in the case of the stacked boxes, or one for each sample set, in the case of the coassemblies. Arrows indicate which files are inputs and outputs for other processes.

**Fig. 3:**
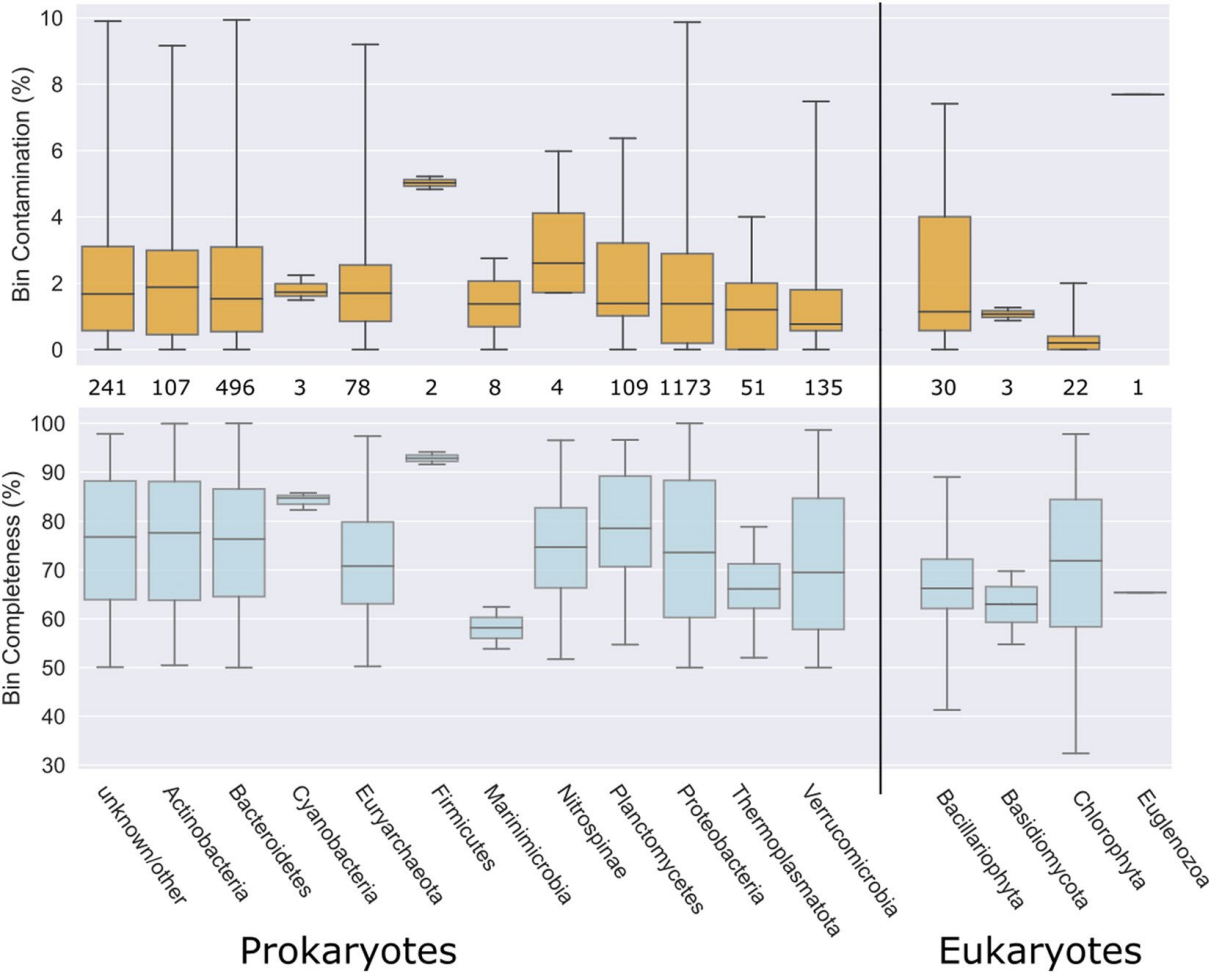
Completeness and contamination of the 2463 MAGs recovered across the 73 samples; 2407 prokaryotic and 56 eukaryotic MAGs. In each panel, a vertical line separates the eukaryotic and prokaryotic MAGs. The number of MAGs per taxon is shown between the two boxplots.

To extract further eukaryotic bins, we used a coassembly method. We used a custom filtering pipeline to identify the eukaryotic fraction of reads from each sample, before pooling these reads for coassembly and binning. To extract the eukaryotic fraction of reads, we used MMSeqs2^26^ (version 0188988235c6f1a8e90f327827c73f981db8a19a) with the default parameters (length cutoff of 500 base pairs) to taxonomically identify contigs that had already been assembled using a per-sample assembly method, using a combination of MMETSP^27^ and NCBI NR^28^ as a reference database. Contigs identified at the domain level as anything other than Bacteria, Archaea, or viruses were retained, leaving a list of putatively eukaryotic contigs. Contigs identified as belonging to already existing prokaryotic bins were removed from this list. Next, the quality-filtered reads were mapped to this subset of contigs with BBMap (v.3.17). These reads were pooled, depending on whether they were from the HAVOC or pilot dataset, and assembled with metahipmer^29^ (version 2.1.0.1.380-gf770aca-dirty-master) for the HAVOC samples, or SPAdes (v3.14.0) with the metaspades.py–only-assembler option for the pilot samples. Samples with no pre-existing metagenome bins from their single assembly were excluded. Finally, the pooled reads from each dataset were mapped to their respective coassemblies, and then the new contigs were binned using MetaBAT 2 (version 2:v2.15-30-g4ec2ab8), and checked for quality with EukCC^30^ (2.1.1, database version 1.1). Bins of over 90% completeness and less than 5% contamination, and with at least 18 tRNA genes, and with 23S, 16S and 5S genes, were designated as high-quality MAGs, those with above 50% completeness and less than 10% contamination were designated medium-quality, and, for the eukaryotic MAGs, those above 30% completeness and less than 10% contamination were retained and designated as low quality, as per Alexander *et al*.^31^. Figure 2 shows a schematic diagram of the bioinformatics pipeline, and an overview of MAG completeness and contamination is provided in Fig. 3.

### Functional and taxonomic annotation

MAGs recovered from single-sample assemblies were annotated using the IMG/M annotation pipeline (versions ranging between 5.0.23 and 5.1.11), using Genemark (v1.05) and Prodigal (V2.6.3)^32,33^ for gene calling, and HMMer^34^ (3.1b2) to combine analyses from the COG, PFam^35^ (v.34), TIGRFAM^36^ (v.15.0), Cath-Funfam^37^ (v.4.1.0), SuperFamily^38^ (v.1.75), and SMART^39^ (01_06_2016) databases. CRISPRs, and tRNAs were identified with CRT^40^ (1.8.2) and tRNAscan-SE^41^ (2.0.4) respectively. Prokaryotic MAGs were taxonomically placed with GTDB-tk (v.2.4.0, database release 220)^42^. GO terms were included based on the PFam2GO mapping provided by Interpro^43,44^.

To identify genes within the coassembled eukaryotic MAGs within the coassemblies, we used MetaEuk^45^ (version f32e8dfc6b994025627326d0f461f3e9903e997e) with the–easy-annotate option, using a custom database of the combined Phycocosm^46^, MMETSP, and UniRef^47^ databases, with UniRef clustered at a 50% identity level. These were combined with genes identified through Genemark-ES (version 4.71_lic, gmes_pepal.pl–ES), with genes from MetaEuk given priority and retained if overlapping with genes from Genemark-ES. PFam (v.35.0), PANTHER (v.17.0), SMART (v.9.0), NCBIfam (12.0) and SuperFamily (v.1.75) domains were then annotated using InterProScan^48^ (version 5.63–95.0).

**Fig. 4:**
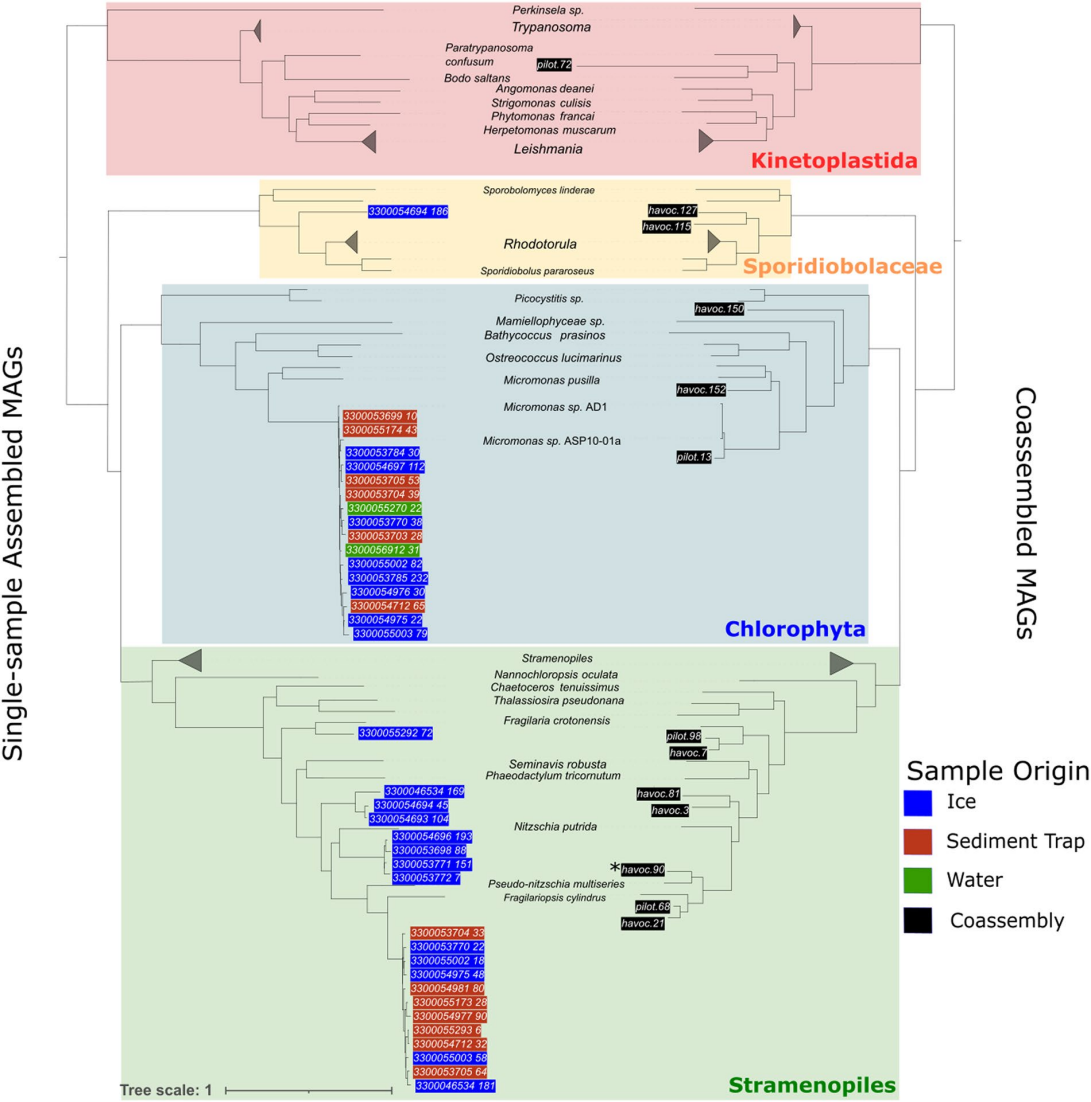
Phylogenetic trees showing MAGs from singly assembled samples (left) and MAGs from coassemblies (right). Reference genomes, common to both trees, have their leaves aligned and are labelled at the centre of the tree, with collapsed clades represented in the tree by a wedge. Some leaves of reference genomes are unlabelled for legibility, where they share their genus with their closest relative in the tree. MAGs labelled are shown on the tips of each tree. For each MAG, the colour of the background indicates the type of the sample (or indeterminate type, in the case of the coassemblies). The coassembled MAG havoc.90, marked with an asterisk *, had good completeness and contamination scores and was subsequently renamed *Bacilliariophyceae sp. MOSAICH1_1*.

Eukaryotic MAGs were placed on a phylogenetic tree (Fig. 4), using a set of 100 concatenated BUSCO^49^ genes (BUSCO v5.1.1 odb_eukaryota_10 gene set, aligned using MUSCLE^50^ v3.8.1551), alongside a set of 140 eukaryotic reference genomes from Phycocosm and NCBI RefSeq^28^. A maximum-likelihood tree was generated using FastTree (2.1.11).

### Case-Study of a high-quality MAG

From our eukaryotic coassembly, we flagged bin havoc.90 from the HAVOC dataset as having good contamination and completeness scores, as measured by BUSCO (v5.1.1, odb_eukaryota_10 gene set), with completeness and contamination of 73% and 0.4% respectively. This MAG, *Bacilliariophyceae sp. MOSAICH1_1*, was annotated using the Phycocosm pipeline; in brief, scaffolds were masked for repeats with RepeatMasker, and genes were called using a combination of Genemark-ES (version 4), GeneWise^51^ (version 1), and fgenesh^52^ (fgenesh1_pg), with the best fitting model for each locus picked to form a set of filtered gene models. Genes were functionally annotated for signal peptides, transmembrane domains, and assigned functional descriptions including assignment of PFam, GO, KOG and KEGG terms, and genes were formed into gene clusters using the MCL algorithm^53^.

This genome had a length of 32.07 Mbp, contained in 2963 scaffolds, with 13169 genes called among the set of filtered gene models. The most closely related isolate genome in Phycocosm was the diatom *Pseudo-nitzschia multiseries* CLN-47, with an average nucleotide identity of 76% (estimated with fastANI v1.33).

## Data Records

All MAGs are available through Figshare^54^, at NCBI BioProject PRJNA1160706 (Data Citation 2)^55^, and replicated in the GOLD database^56^ (https://gold.jgi.doe.gov), Study ID 505419. Annotations of *Bacilliariophyceae sp. MOSAICH1_1* are also available through the Phycocosm web portal (https://phycocosm.jgi.doe.gov/Mosaich1_1/Mosaich1_1.home.html), and via Figshare.

Individual read files for samples are stored in the NCBI SRA, with BioSample, BioProject, and SRP accessions listed in Supplementary Table 1, and as citations^57–129^.

## Technical Validation

Prokaryotic MAG completeness and contamination was estimated using CheckM^25^, and only those MAGs within the MIMAG standards of 50% completeness and below 10% contamination were retained. Eukaryotic MAG completeness and contamination was estimated using EukCC – those above 30% completion and less than 10% contamination were retained, with those under 50% completion designated as low-quality MAGs.

## Supplementary information

Supplementary Table 1

## Data Availability

The custom pipelines used for eukaryotic MAG binning and annotation are available at https://github.com/willboulton/mosaic-pilot-havoc-mags.
